# Prosthetic Joint Infection due to *Mycobacterium bovis* after Intravesical Instillation of Bacillus Calmette-Guerin (BCG)

**DOI:** 10.1155/2009/527208

**Published:** 2009-12-16

**Authors:** Eric Gomez, Tom Chiang, Ted Louie, Madhavi Ponnapalli, Robert Eng, David B. Huang

**Affiliations:** ^1^Division of Infectious Diseases, New Jersey Medical School, University of Medicine and Dentistry of New Jersey (UMDNJ), Newark, NJ 07101, USA; ^2^Veteran's Affairs New Jersey Healthcare System, 385 Tremont Avenue, 111-ID, East Orange, NJ 07018, USA; ^3^Allergy, Immunology, and Infectious Diseases, Robert Wood Johnson University Hospital, New Brunswick, NJ 08903, USA

## Abstract

Intravesical instillation of Bacillus Calmette-Guerin (BCG) is a treatment to prevent recurrence of superficial urothelial bladder carcinoma. Complications after bladder instillation of BCG have been reported including locally invasive and systemic infections due to dissemination of *Mycobacterium bovis* from the bladder. We present an uncommon case and literature review of prosthetic joint infection due to *M. bovis* after intravesical BCG treatment of bladder cancer.

## 1. Introduction

The Bacillus Calmette-Guerin (BCG) has been used as intravesical immunotherapy for superficial urothelial bladder carcinoma in preventing its recurrence [1]. However, this modality of treatment is not benign and may have complications. Side effects range from locally and self-limited complications to systemic and life-threatening adverse reactions [2]. Osteoarticular complications after intravesical BCG instillation can present as infectious and noninfectious, the latter being the most common [3]. An infectious osteoarticular complication of BCG, which implies invasive *M. bovis* disease, is rare. We report a case of invasive BCG disease causing prosthetic hip infection in a patient, 19 months after intravesical BCG instillation for superficial bladder carcinoma. Other cases of prosthetic joint infection due to BCG after treatment of bladder cancer were also reviewed.

## 2. Case Report

**Figure 1:**
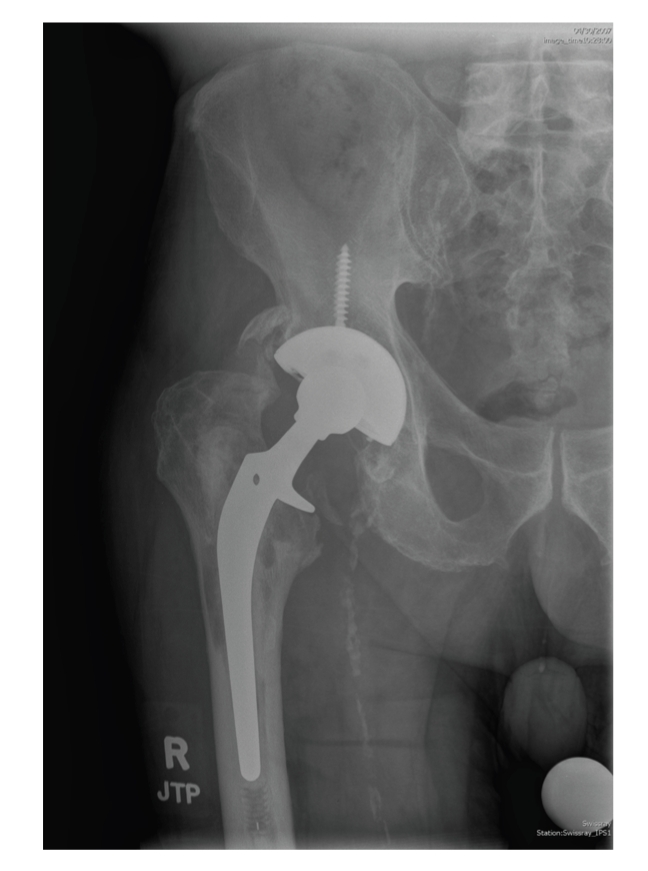
Anteroposterior X-rays of right hip showing lucencies around femoral and acetabular prosthesis.

In June of 2007, an 82-years-old man with a history of right total hip arthroplasty (THA), in 1997 due to osteoarthritis, presented to his primary care physician (PCP) with right hip pain. His past medical history was significant for a diagnosis of papillary urothelial carcinoma of the bladder in June 2005 and a subsequent transurethral bladder resection and BCG bladder instillation from July 2005 to October 2005. He received a total of 6 weeks of BCG instillation at 50 mg per dose, divided in two cycles of three weekly doses separated by one month. The initial workup for the presenting right hip pain showed loosening of hardware by imaging (Figure 1). He denied fever, chills, night sweats, or weight loss. His physical exam was unremarkable. His white blood cell count was 8.8 × 10^3^/*μ*L (normal: 4.5–11 × 10^3^/*μ*L) with 75% neutrophils, and his hemoglobin was 10.8 g/dL (normal: 13.8–18 g/dL) with mean corpuscular volume of 92.2 fL (normal: 80–95 fL). He underwent exploration of his right THA and purulent material (drainage) was found in the joint. Intraoperative Gram stain was negative and the THA was replaced. THA Gram stain showed rare leukocytes, and multiple aerobic and anaerobic bacterial cultures were negative. Acid fast bacilli (AFB) stain and culture were not done on this initial exploration and THA replacement. Erythrocyte sedimentation rate (ESR) levels were not done preoperative; however, postoperative ESR levels were 51 mm/60 minutes (normal: 8–15 mm/60 minutes). The patient was treated for culture negative septic arthritis with eight weeks of ertapenem 1 gram IV daily. In December 2007, his right hip became indurated and tender. One month later he developed a soft and erythematous raised lesion at the site of the surgical wound. Amoxicillin-clavulanic acid 875/125 mg orally twice daily was prescribed for 2 weeks by his PCP with no improvement.

In March 2008, a second right THA revision was performed with the suspicion of a superficial surgical wound infection. However, purulent fluid was found tracking down from the surgical wound to the THA. Irrigation, debridement, and replacement of the femoral head were done. Again, intraoperative cultures of the hip were negative. Ertapenem 1 gram IV daily was empirically started. Intraoperative AFB stain from the right hip prosthesis was negative; however, the mycobacteria culture was reported positive for growth on day 39 of incubation. *Mycobacterium tuberculosis* complex was identified by DNA probe (Gen-Probe, San Diego, CA, USA). Isoniazid, rifampin, ethambutol, and pyrazinamide were empirically started. No risk factors for tuberculosis were identified. His chest X-rays were unremarkable; a PPD was not done. Based on the history and due to the high suspicion of *M. bovis* BCG infection, the isolate was sent to a reference laboratory for speciation. Using polymerase chain reaction methodology, *Mycobacterium bovis* was identified and a deletion of the RD1 region was noted confirming *M. bovis* BCG strain. Susceptibility testing showed resistance to pyrazinamide only. Pyrazinamide and ethambutol were discontinued and rifampin and isoniazid were continued. Since the patient was deemed high surgical risk to undergo THA revision, the patient was treated with antibiotics only and to be closely followed up for any possible relapse. After 12 months on treatment for *M. bovis*, the patient continues to improve without signs of active infection in right THA.

## 3. Discussion

Calmette and Guerin started to develop tuberculosis vaccine in 1908, and after multiple passages of virulent *Mycobacterium bovis* was attenuated [1]. In 1921, the BCG vaccine was launched for immunization against tuberculosis in humans. Soon it became evident that the BCG vaccine had immunomodulating effects in cancer patients. However, it was not until 1976 when it was used clinically to treat a patient with bladder cancer [1]. Since then, BCG has been established as a routine immunomodulating agent for superficial bladder cancer and one of the treatments in prevention of recurrent disease [4].

BCG bladder instillation can cause local and systemic side effects. After instillation, BCG will cause a local inflammatory reaction that can manifest as fever and hematuria. These symptoms are self-limited and typically improve with symptomatic treatment. Systemic adverse reactions due to BCG bladder instillation can range from granulomatous prostatitis to BCG sepsis with high mortality if not treated [5]. 

Lamm et al. reported a review of complications after BCG intravesical treatment of 2602 patients with bladder cancer [6]. The most common complication was cystitis, followed by fever and granulomatous prostatitis. Systemic BCG infection defined as pneumonitis or granulomatous hepatitis occurred in 0.7% of patients who received intravesical BCG. BCG sepsis was seen in 0.4% of patients with an estimated mortality of 1 death per 12,500 patients. 

Musculoskeletal complications from intravesical BCG were reported to be rare by Lamm et al. [6]; however, multiple cases have been reported over the years. The most common complications included arthralgia and arthritis which some authors have reported their occurence in 0.5 to 1% of patients [3]. This complication is thought to be a reactive arthritis due to a systemic response of the host to the BCG and not a direct invasion of the joint by the attenuated *M. bovis*. Cultures are usually negative and the symptoms usually respond to anti-inflammatory treatment.

Invasive BCG infection of muscle and bone structures (with positive cultures) is rare but has been reported in literature. Clavel et al. reviewed eight cases of *M. bovis* osteoarticular infections after intravesical BCG including septic arthritis, prosthesis joint infections, and discitis [7]. 

*M. bovis* arthroplasty infection without a history of BCG exposure has been reported but it seems to be exceeded by intravesical BCGs related prosthetic joint infections [8]. A review of literature reveals four other cases of *M. bovis* prosthetic joint infections (Table 1). Patients had an average age of 77 years at presentation, and they all had prosthetic joint procedures 6 to 12 years prior to the BCG treatment. Most patients presented with subacute to chronic joint pain. For the majority of the patients, the presumptive diagnosis was aseptic prosthetic failure. Only one patient presented with signs of acute infectious process with rigors and sweats [9]. 

Prosthetic joint infections can occur anytime from 7 days to years after the intravesical treatment with BCG. Early presentation of infection could be due to hematogenous dissemination of *M. bovis* bladder instillation. This has been minimized by delaying treatment for at least 2 weeks after surgical resection of the bladder tumor and by avoiding traumatic catheterization of the bladder [2]. 

Explaining the acquisition of *M. bovis* prosthetic infections months after the treatment poses a challenge. This may be explained by the persistence of mycobacteria in the bladder with subsequent dissemination months after treatment. Some studies have shown that 6 weeks after treatment there are no viable mycobacteria in the bladder [13]. However, Bowyer et al. found that five out of 90 patients had persistent mycobacteria in the urine up to 16.5 months after intravesical BCG treatment [13]. Breaches in the mucosal and immunologic barrier can then occur months after treatment.

Systemic persistence of *M. bovis* with later reactivation and seeding of the prosthesis could account for the late presentation of our patient. Böhle et al. reported a case of BCG pulmonary infection 11 months after radical cystectomy in a patient with bladder cancer treated with intravesical BCG [14]. The authors proposed early dissemination of BCG from the bladder, seeding the lungs and reactivation months later. This also has been seen with intradermal BCG presenting years later with lymphadenitis.

Treatment of BCG prosthetic joint infection is anecdotal and not well defined. Based on case reports of tuberculosis infection of arthroplasties, Marmor et al. reported successful treatment with antituberculous drugs along with surgical procedures [15]. Antituberculous regimen included at least 3 drugs ranging from 6 to 8 months. Most cases require a two stage revision arthroplasty along with chemotherapy. One stage revision arthroplasty and even retention of prosthesis with synovectomy and debridement have been reported with good outcomes [16]. Spinner et al. found that late onset of tubercular prosthesis infections often tend to fail with retention of the hardware [17].

From the review of the literature (Table 1), three out of four patients with *M. bovis* prosthetic joint infections were treated with two stage arthroplasty replacement, with one treatment failure. The treatment failure was noted to have persistent infection by pathology but negative cultures after six months of antituberculous drugs treatment [12]. Two patients underwent a one-stage arthroplasty replacement with good outcome, including our patient [10].

Antituberculous drugs used to treat BCG infections include isoniazid, rifampin, ethambutol, and cycloserine. *M. bovis* has been noted to have intrinsic resistance to pyrazinamide in vitro, and therefore, this drug is never recommended in the treatment of BCG infections. Isoniazid, rifampin, and ethambutol suppressed mycobacterial growth by day 7 of treatment, and cycloserine inhibits growth of *M. bovis* by 24 hours in animal studies [2]. However, a recent study showed increased cycloserine resistance in BCG *M. bovis* strains [18]. As most BCG prosthesis infections presented in a subacute/chronic course, treatments would typically include a four-drug regimen with first-line antituberculous drugs followed with deescalation to two or three drugs once *M. bovis* has been identified by culture.

Duration of treatment has also varied in the different case reports. On average, patients receive a minimum of one-year treatment. Treatment of less than one year may lead to failure as in the case reported by Guerra et al. [12]. Failure was determined by histopathology showing granulomas containing fluorescent bacilli although mycobacterial cultures were negative.

Patients with prosthetic joint infections and bladder cancer treated with intravesical BCG may be at risk for *M. bovis* prosthetic joint infection. Some authors suggest the use of antituberculous prophylaxis in patients with indwelling prosthetic devices who undergo intravesical BCG treatment [19]. However, due to few cases of indwelling prosthetic infections with *M. bovis* after intravesical BCG instillation, the benefits of prophylaxis remain uncertain. Which drug to choose for prophylaxis also remains uncertain.

*M. bovis* prosthetic hip infection after BCG intravesical instillation is a rarely reported condition. Medical and/or surgical treatments need to be further investigated and clarified. With the increase in the aging population, the likelihood patients with prosthetic joints being treated with BCG instillation should alert the physician of the possibility of this infection.

## Figures and Tables

**Table 1:** Case reports of *M. bovis* prosthetic joint infections after intravesical BCG instillation in patients with bladder cancer.

Case report	Age/sex	Orthopedic procedure	Bladder cancer (months PTP)	BCG strain and duration of bladder cancer treatment	Clinical presentation	Cultures/drug susceptibilities	Treatment/condition
Our patient	82, male	Right THR	20	Tice BCG, 6 weeks	Right hip pain	*M. bovis*; sensitive to INH, RIF, ETM. Resistant to PZA	1-Stage THR, INH/RIF × 1 year. Alive and free of symptoms at 1 year

Reigstad and Siewers (2008) [10]	86, male	Left THR	10	Oncotice BCG, 9 weeks	Aseptic loosening of hardware	*M. bovis*; sensitive to INH, RIF, PZA	1-stage THA revision, INH, RIF, PZA × 6 months, INH/RIF × 6 months, and INH × 1 year of consolidation

Segal and Krauss (2007) [11]	76, male	Left THR	48	Multiple treatments. Strain NR	Groin pain	*M. bovis*; susceptibilities unknown	INH/RIF/ETM × 1 year. 2-stage arthroplasty. Alive and free of symptoms.

Guerra et al. (1998) [12]	66, male	Right THR	12	Tice BCG, 12 weeks, radical cystectomy	Hip pain, rigors and sweats	*M*. *bovis*; susceptibilities unknown	INH/RIF × 6 months and 2-stage arthroplasty. Patient died due to his underlying condition.*

Chazerain et al. (1993) [9]	77, male	Left TKR	2.5	Pasteur BCG	Acute arthritis	*M. bovi*s; susceptibilities unknown	2 antituberculous drugs (NR) × 2 years and 2-stage arthroplasty. Alive and free of symptoms.

Abbreviations: prior to presentation (PTP), total hip replacement (THR), total knee replacement (TKR), not reported (NR), isoniazid (INH), rifampin (RIF), and ethambutol (ETM).

∗Death due to lung cancer.
